# Children with Dyslexia Benefit from Short Combined Reading and Motor Training: Objective Measures Assessed by Eye Movements and Postural Sway Recordings

**DOI:** 10.3390/brainsci15111218

**Published:** 2025-11-13

**Authors:** Simona Caldani, Elie Khoury, Richard Delorme, Maria Pia Bucci

**Affiliations:** 1 ICAR UMR 5191, CNRS, ENS de Lyon, Université Lyon 2, 69342 Lyon, France; simona.caldani@gmail.com; 2 Equipe InDev, NeuroDiderot, Robert Debré Hospital, 75019 Paris, France; 3 Child and Adolescent Psychiatry Department, Robert Debré Hospital, APHP & Université Paris Cité, 75935 Paris, France; elie.khoury@aphp.fr (E.K.);; 4 Human Genetics and Cognitive Functions, Institut Pasteur, 75015 Paris, France

**Keywords:** dyslexia, children, reading, posture, training, cerebellum

## Abstract

**Background/Objectives:** Children with dyslexia report poor motor control; several studies have shown poor eye movements control during reading and important body instability in these children. The present study aimed to test in children whether reading and postural abilities in children with dyslexia could benefit from a short combined reading and postural training program. **Methods:** Thirty-two children with dyslexia were randomly assigned to training group (G1) or control group (G2). All participants completed eye movements recording during reading and postural recording under an unstable support before and after the intervention. G1 underwent a 10 min combined reading and postural training while G2 had a 10 min rest. During reading, the reading time, the duration of fixations, as well as the occurrence, amplitude, and number of forward saccades (saccades from the left to the right) and backward saccades (saccades from the right to the left) were measured. The PII (postural instability index) was measured under unstable support. G1 exhibited a significant decrease after training in reading time, fixation duration, and the number of forward saccades. Furthermore, we observed a significant reduction in postural instability. In contrast, G2 failed to show any significant changes in eye movements and postural recordings. **Conclusions:** We suggest such a combined reading and postural training approach could help dyslexic children to improve motor abilities. Adaptive mechanisms through improved cerebellar activity could be responsible for such motor enhancement.

## 1. Introduction

Dyslexia is a difficulty in learning to read accurately and fluently despite adequate intelligence and practice, without the presence of any sensory or neurological disorders, with normal school instruction and sufficient sociocultural opportunities [1]. The America’s Children and the Environment (ACE) (www.epa.gov/americaschildrenenvironment, accessed on 10 November 2025) recently reported that dyslexia occurs in 10.8% in children and adolescents (5–17 y.o.). Dyslexia is due to a phonological disability, notably the difficulty in applying grapheme–phoneme conversion rules between letters and their corresponding sounds in the language. This deficit is considered one of the best-known characteristics of dyslexia and is linked to insufficiently specified phonological representations [2,3]. However, other hypothesis have been advanced for explaining the origin of dyslexia (see review of Stein [4]. Researchers have reported disabilities in auditory perception [5], impaired working memory skills [6], attentional abnormalities [7], and visuo-attentional immaturities [8]. Nicolson et al. [9] also advanced the hypothesis that a cerebellar deficit would lead to dyslexia, based on insufficient integration of sensorial inputs leading to balance impairment and poor skill automatization. Several studies reported postural instability in dyslexic children [10,11,12,13], reinforcing the cerebellar deficit theory.

Nicolson & Fawcett [14] suggested that a cerebellar abnormality could be the origin of both motor as well as articulation disorders. The lack of articulatory fluency could lead to an impoverished representation of the phonological characteristics of speech and subsequently, to difficulties with phonological awareness, leading in deficits in learning to read. Difficulties in reading, spelling, and writing may also result from deficits in motor automaticity. Such deficits are also observed in eye movements performance, particularly during reading in children with dyslexia. Indeed, even if it is not recognized by all the scientific community, several studies have reported that dyslexic children show an abnormal oculomotor pattern during reading (see review from Premeti et al. [15]). Briefly, several researchers reported abnormal eye movements in dyslexic children: several forward (from left to right) and backward (saccades from right to left) saccades, and several fixations of longer duration. Other studies have also shown significant instability during fixation. Note that such oculomotor pattern is also present in dyslexic children of different countries, suggesting that it occurs irrespective of the orthography of the language [16,17,18,19,20,21,22,23,24,25]. This oculomotor pattern is characteristic of dyslexic children, while non-dyslexic children, for whom reading skills are well developed, show shorter fixation durations and smaller saccade amplitude. The abnormal oculomotor pattern reported in dyslexic children is most likely due to immaturity of cortical structures responsible for controlling both eye movements and reading processing [26].

Many researchers have developed new training techniques to improve reading deficits in children with dyslexia [27,28,29]; in contrast, few studies have tested reeducation therapies involving postural and cognitive abilities at the same time. Our group [30] was, to our knowledge, the first to show that a short postural training period (3 min) improved posture in dyslexic children. Ramezani et al. [31] tested a new Verbal Working Memory–Balance (VWM-B) program combining memory and balance training using a robotic platform for children with dyslexia. Training of working memory and postural skills was performed for five weeks (three sessions/week/45–60 min per session). These authors reported improvements in both verbal working memory and postural capabilities in these children, supporting the cerebellar deficit theory in dyslexia. Interestingly, in a subsequent fMRi study in some of these children [32], these authors aimed to estimate the functional connectivity alterations after 15 sessions of training by the VWM-B. They reported a significant increase in functional connectivity in the left fusiform gyrus and specific parts of the cerebellum (left and right Crus II) after the training. This finding suggests the occurrence of adaptive mechanisms in these cortical structures.

The goal of the present study was to test a training program (of 10 min) in which children with dyslexia were trained simultaneously to read words and control their posture. Our driving hypothesis was that adaptive mechanisms through a better cerebellum activity could improve both reading skills and postural sway. These abilities have been objectively assessed by using an unstable platform for measuring postural sway and an eye tracker to measure oculomotor pattern during reading of a text.

## 2. Methods

### 2.1. Participants

The minimum required sample size was determined using the G*power [33] software was used to determine the minimum sample size. Based on prior research [34], the α and power (1 − β) values were set at 0.05 and 0.8, respectively. To ensure sufficient statistical power and minimize the risk of a type 2 error, data from twenty-two participants were deemed necessary.

Two groups (G1 and G2), each consisting of sixteen dyslexic children, were tested (see Table 1). Children were recruited at the Robert Debré pediatric university hospital (Paris, France). The allocation of a child to a specific group (experimental group, G1 or control group, G2) was randomly selected. Group G1 only benefited from combined reading and postural rehabilitation.

All children underwent a neurological examination and children with psychotropic treatment and/or with psychiatric comorbidities (such as ADHD, ASD) were excluded. The Wechsler Intelligence Scale for children (WISC-V) was used to assess their cognitive skills, with all participants having mean intelligence quotient (IQ) in the normal range (between 85 and 115). The L2MA (Langage Oral Écrit Mémoire Attention) battery by Chevrie-Muller et al. [35] was used to evaluate dyslexia. Note that this French battery is used by clinicians to test oral and written language abilities (text comprehension, ability to read words and pseudo-words) and memory capabilities. All children had a score of more than two standard deviations below the normal mean on the L2MA. Reading age for children was evaluated with the ELFE test (Évaluation de la Lecture en FluencE) (www.cognisciences.com, Grenoble, France), where children read aloud a text for 1 min and the number of words read was measured.

**Figure 1:**
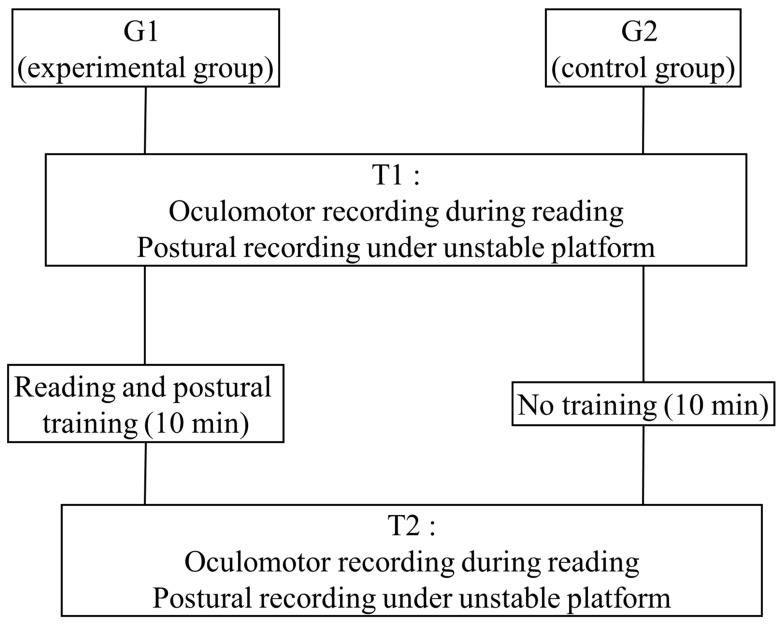
Description of the trial design.

Eye movements during reading and postural control were recorded at T1 and T2, respectively, i.e., before and after 10 min of reading and postural training for the experimental group (G1), and before and after 10 min of rest (i.e., without reading and postural exercises) for the control group (G2). During this period of 10 min, children of the G2 were free to speak with the experimenter. Figure 1 shows the trial design. The experimenter who analyzed the data was blinded to which group the child was in.

The investigation followed the principles of the Declaration of Helsinki and was approved by our Institutional Human Experimentation Committee (Comité de Protection des Personnes CPP Île-de-France I, 2021-A00489-32, 11 May 2021). Written consent was obtained from the children’s parents after the experimental procedure was explained to them.

### 2.2. Oculomotor Recording

Eye movements were performed with an eye tracker with infrared cameras with a recording frequency of 300 Hz. Movements of each eye are recorded independently with a precision of 0.25°. The experiment was run in a dark room, with the child seated 60 cm from the front of a screen and the head stabilized. Viewing was binocular. Calibration was performed at the beginning, and the child had to fixate 13 points of 0.5 deg on the screen. Each calibration point required a fixation of 250 ms to be validated. A polynomial function with five parameters was used to fit the calibration data and to determine the visual angles. After this calibration, the reading task started. The duration of the reading task was kept short (few minutes) for an accurate evaluation of eye movement recordings [26].

The child had to read aloud four lines of a text extract from a children’s book (extract from Jojo Lapin Fait des Farces, Gnid Bulton, Hachette) [26,36]. Two different texts were used for T1 and T2 oculomotor recordings (for details, see Caldani [36]); the order of the text in T1 and T2 was counterbalanced. It should be noted that the child was instructed to read aloud the text. After the reading task, three questions were asked in order to verify the comprehension of the text read.

### 2.3. Postural Recording

The Multitest Equilibre (www.framiral.fr) medical device was used to measure posture [13]. The surface of the center of pressure displacement (CoP) was recorded on an unstable platform under three different viewing conditions: eyes open (EO), fixating a target (0.5°) projected on a screen at 250 cm in front of the children at their eye level; eyes closed (EC); and eyes open with optokinetic stimulation (perturbed vision, (OPTO), see Gouleme et al. [37]). The displacement of the Center of Pressure (CoP) was sampled at 40 Hz and digitized with 16-bit precision. Postural recording was performed in a dark room. While the child stood on the platform, the child was instructed to stay as still as possible with their arms along the side of the body and the feet position standardized on footprints (distance and angle between heels: 11 cm and 30°, respectively). Each postural condition lasted 30 s, with 15 s of rest to avoid effects from fatigue. Postural capability on an unstable support under three different visual conditions (EO, EC, and OPTO) was recorded at T1 and T2, with the order of three visual conditions counterbalanced across participants.

### 2.4. Combined Reading and Postural Training Protocol

The child was on an unstable support of the Multitest Equilibre and had to read in front of them single words that were projected (viewing distance of 2.5 m). The child was asked to control his/her balance and at the same time to read aloud the word that was located at a different position in front of him/her (on the right or left side, up or down). After reading the word correctly aloud, another word was presented to the child. The length of the first word was short (of four letters) and after of six, seven, or eight letters. This type of training lasted 10 min. Words were written in Arial, and each letter was 0.5 deg. Ten words of four, six, seven, and eight letters were used and they were extracted from the French database Lexique 3 [38].

### 2.5. Data Analysis

For the oculomotor parameters during reading, we measured the total reading time, the duration of fixations, the number and amplitude of forward and backward saccades.

For assessing postural performance, we measured the postural instability index (PII) (see [15]. The PII is calculated as follows: PII = Σx Σy PI (F1, F2, F3)/CT (F1, F2, F3), where x is the mediolateral and y is the anteroposterior direction; PI and CT are the spectral power index and canceling time, respectively, for different frequency bands (F1: low frequency between 0.05 and 0.5 Hz; F2: medium frequency, between 0.5 and 1.5 Hz; and F3: frequency higher than 1.5 Hz) [39]. The PI is calculated as the decimal logarithm and allows us to know the postural oscillation and the energy cost involved to maintain postural control. CT is the time, in seconds, during which the spectral power index of the body sway was almost canceled by postural control mechanisms. Thus, using the PII is a relevant choice to report postural performance in children [37]. Note that this parameter is a global postural index used during routine tests by clinicians; a higher PII means greater body instability.

### 2.6. Statistical Analysis

Normality (Shapiro–Wilk test) and homogeneity of variance (Levene test) were tested before running ANOVA. We performed a one-way ANOVA with the two groups of children (experimental group, G1 and control group, G2) as the between-subject factors, and clinical characteristics of children as the within-subject factors. Repeated-measures ANOVA was run between the two groups of children (G1 and G2) as the between-subjects factor for each oculomotor variable during the reading task, recorded twice (Two levels: T1 and T2) as the within-subjects factor. Repeated-measures ANOVA was also run between the two groups of children (G1 and G2) as the between-subjects factor on the postural instability index recorded at two time points (Two levels: T1 and T2) during the three distinct visual conditions (three levels: EO, EC, and OTPO) as the within-subjects factor. Data were processed using JASP software version 0.16.4.0 (University of Amsterdam). The Bonferroni test was used for post hoc comparisons. η2 values are reported, and a 95% confidence interval was applied. The difference was considered significant when the *p*-value was less than 0.05.

## 3. Results

**Figure 2:**
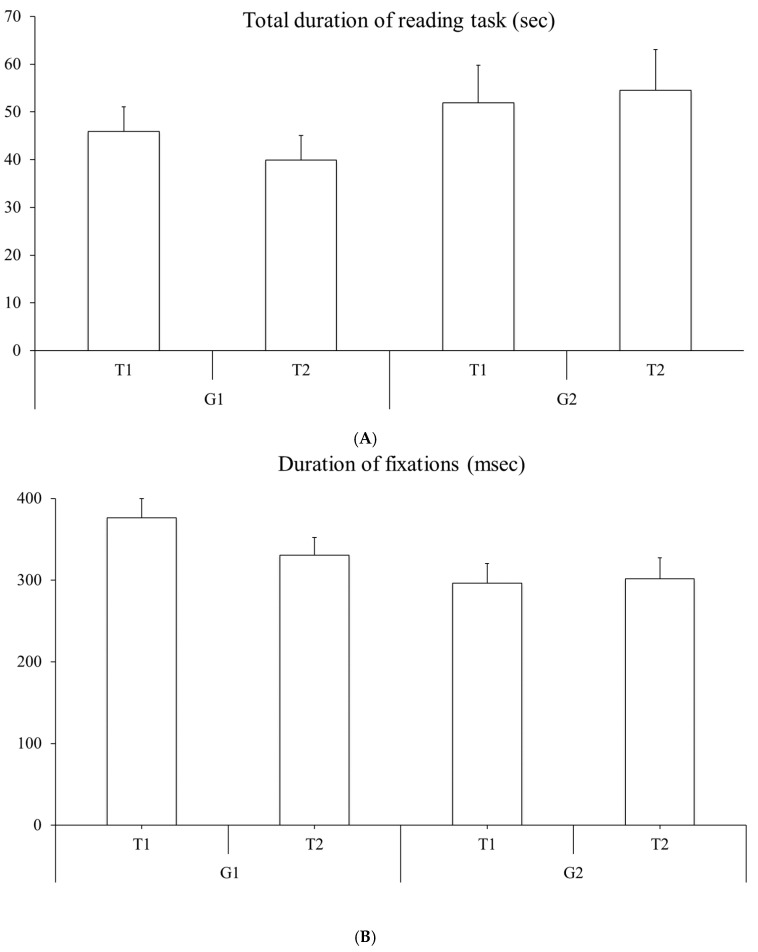
Oculomotor parameters recorded during reading in T1 and T2 for the two groups of children tested. (**A**) Mean and standard error of total reading duration (s) and (**B**) durations of fixations (ms).

Eye movements measured during reading. Figure 2A shows the total reading time measured at T1 and T2 for the two groups of children (G1 and G2). ANOVA reported a significant interaction T x G effect (F_(1,30)_ = 8.049, *p* = 0.008, η2 = 0.06). The Bonferroni post hoc test revealed that the reading time was significantly shorter in the group G1 at T2 with respect to T1 (*p* = 0.05, Cohen’s d = 0.218).

The duration of fixations is shown in Figure 2B. ANOVA showed a significant T x G interaction effect (F_(1,30)_ = 5.500, *p* = 0.026, η2 = 0.08). The Bonferroni post hoc test revealed that the duration of fixation was significantly shorter in the group G1 at T2 with respect to T1 (*p* = 0.03, Cohen’s d = 0.472). Mean and standard errors of the total duration of reading task and durations of fixations for the two groups are reported in Table 2.

The percentage of correct responses at T1 and T2 for the two groups of children was also evaluated. At T1, the comprehension values were 78% and 61%, for G1 and G2, respectively, while at T2 they reached 93% and 57% for G1 and G2. Although comprehension was evaluated by three questions only for each text, ANOVA reported a significant T x G interaction effect (F_(1,30)_ = 14,154, *p* < 0.001, η2 = 0.03), with a significantly higher comprehension for G1 at T2 (*p* = 0.002, Cohen’s d = 0.566).

**Table 3:** Mean and standard error of the number and amplitude (deg) of forward and backward saccades and their standard errors during reading recorded at T1 and T2 in the two groups of children tested. Asterisk indicates a significant difference in the Bonferroni post hoc test.

Forward Saccades:				
	T1		T2	
	Number	Amplitude (deg)	Number	Amplitude (deg)
**G1**	66 ± 1	2.75 ± 0.20	52 ± 2 *	2.76 ± 0.28
**G2**	70 ± 7	2.98 ± 0.33	72 ± 9	2.90 ± 0.34
**Backward saccades:**				
**G1**	24 ± 4	2.69 ± 0.19	19 ± 3	2.85 ± 0.14
**G2**	25 ± 8	2.79 ± 0.29	26 ± 7	2.98 ± 0.16

### Postural Measures

**Figure 3:**
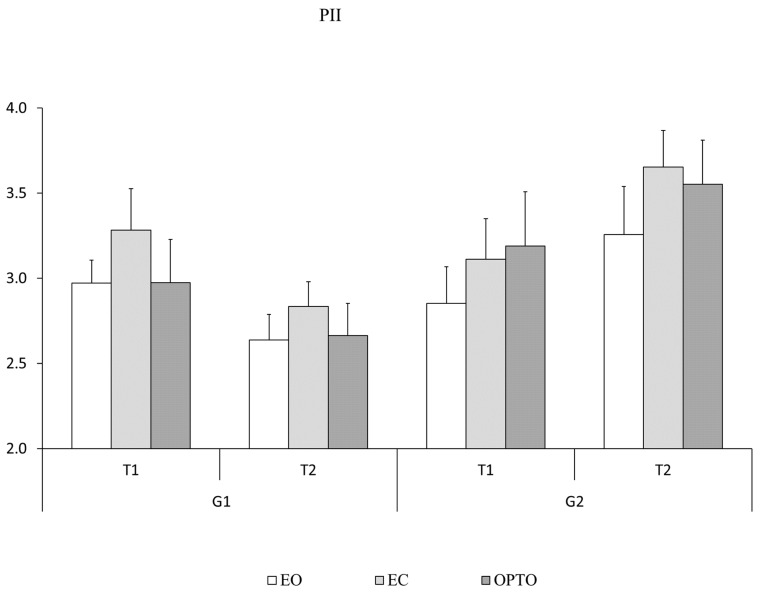
Postural parameter. Mean and standard error of the postural instability index measured in T1 and T2 for the two groups of children tested in the different visual conditions (eyes open, EO, eyes closed, EC, and perturbed vision, OPTO).

The Postural Instability Index (PII) was measured in the three visual conditions (EC, EO, and OPTO) at T1 and T2 for the two groups of children tested (Figure 3). ANOVA showed a significant T x G interaction effect (F_(1,30)_ = 26.185, *p* < 0.001, η2 = 0.09) with a PII that was significantly smaller at T2 than at T1 (*p* < 0.013, Cohen’s d = 0.310) in the G1 group only. We also observed that PII’s values depended on the visual inputs (F_(2,30)_ = 3.422, *p* < 0.03, η2 = 0.08, Cohen’s d = 0.318) with a significant reduced stability in the EC than in the EO conditions (*p* < 0.03).

## 4. Discussion

The goal of the present study was to explore the benefits of a short period of combined reading and postural training on both reading and postural skills in dyslexic children. We reported that 10 min of this training improved oculomotor and postural skills in children with dyslexia. More specifically, concerning reading skills, we reported a significant reduction in reading time, fixation duration, and the number of forward saccades. Furthermore, we observed a significant reduction in postural instability. Interestingly, and in line with previous studies, we reported that visual input is the principal sensory channel for achieving good body stability.

### 4.1. Oculomotor Performances

In the literature, several studies have reported beneficial effects of visual attention and/or oculomotor training on reading skills in dyslexic children. For example, Peters and colleagues [40], reviewing 18 studies, observed improvement in reading skills after visual perceptual training in dyslexic individuals, allowing an enhancement in reading fluency, comprehension, and reading accuracy. Our group also showed that, after a short visuo-attentional training, children with dyslexia improved their reading skills [36]; they learned to read faster, with shorter duration of fixations. Van der Stappen et al. [41] also reported that rapid automatized naming (RAN) training in dyslexic individuals improved reading performance and increased activity in dorsal cortical structures, including the left anterior segment of the arcuate fasciculus, that are linked to reading abilities. In the present study, we reported improvements concerning reading skills suggesting that reading functions, when trained, can contribute to the development of motor skills necessary for efficient reading.

### 4.2. Postural Control

In line with other findings, children with dyslexia showed postural instability and motor coordination deficits, suggesting cerebellar impairment [30,42,43,44,45,46,47]. Postural control requires complex activity between sensory and motor information [45]; it is well known that cerebellum plays a pivotal role in coordinating sensory and motor inputs. In our study, we found that postural training had a beneficial effect on postural control in children with dyslexia. This improvement may be related to a more efficient use of muscular patterns to achieve postural stability, possibly supported by brain plasticity, which enhances both sensory processing and cerebellar integration.

### 4.3. Effect of Combined Reading and Postural Training Protocol

There are not a lot of studies that propose to investigate the combined effects of cognitive and postural performance simultaneously in dyslexia. As mentioned in the introduction, Ramezani et al. [31] reported that dual-task training (cognition and motor skills) led to an improvement in both verbal working memory and postural capabilities in dyslexic children. These authors suggested a relationship between postural and cognitive capabilities. More recently, these same authors [48] reported that this type of training could improve several executive functions such as attention, inhibition, working memory, processing speed, naming ability, and capability to lexical access in dyslexic children. More recently, Ben Dhia et al. [49], exploring the effect of combined cognitive and motor training in Tunisian dyslexic children reported improvements in reading, writing, and motor coordination. These authors suggested that this type of training could improve cerebellar activity, thereby improving reading, writing, and motor skills in dyslexic children. In fact, it is well known that the cerebellum is important not only for controlling motor activities, but also for other cognitive tasks such as attention, working memory, and action planning [50,51]. The cerebellum is also important for the development of cortical structures such as the frontal and parietal cortices [52]. In light of our findings, we propose that combined cognitive and motor training may provide stimulation that enhances cerebral plasticity, allowing better cognitive and motor functions in children with dyslexia.

## 5. Limitations

This study has several limitations. Firstly, the limited number of subjects tested; other studies on a population of dyslexic subjects are needed to confirm these results. It would also be interesting to compare the trained group with a well-matched control group. Secondly, imaging studies are necessary to explore the cortical/cerebellar structures responsible for these improvements. We also have to point out that in the present study, articulation disorders (due to cerebellar deficiencies [52]) have not been tested. Further studies examining the effect of cognitive and motor training are needed to test articulation capabilities in dyslexic children. Finally, a follow-up study will be useful in order to evaluate whether reading and postural abilities are stabilized over time.

## 6. Conclusions

Our results underline the importance of reeducation that combine postural and reading exercises, which could allow children with dyslexia to improve motor functions, even if there are several methodological constraints (control design, short-term testing, sample size). We can suggest that even such a short period of training could promote functional reorganization of cerebellar–cortical network, thereby improving performance across both cognitive and sensorimotor domains. These hypotheses need to be confirmed by further studies on a larger number of children.

## Figures and Tables

**Table 1:** Clinical characteristics of the two groups (experimental group, G1 and control group, G2) of children with dyslexia enrolled in the study.

	G1 Experimental Group N = 16 Children	G2 Control Group N = 16 Children
Age (year)	10 ± 0.28	10.9 ± 0.60
Sex (F/M)	2/14	3/13
ELFE test (words/min)	64 ± 8	66 ± 10
**L2MA standard deviation from the mean:**		
Oral language	2.7	2.6
Written language	2.8	2.7
Memory	2.5	2.6
**Wechsler scale (WISC-V) scores:**		
Verbal Comprehension Index	97 ± 4	98 ± 5
Visual Spatial Index	90 ± 6	91 ± 4
Working Memory Index	95 ± 3	96 ± 5
Fluid Reasoning Index	98 ± 5	96 ± 4
Processing Speed Index	96 ± 6	97 ± 7

**Table 2:** Mean and standard error of total reading duration (s) and durations of fixation (ms) for the two groups of children at T1 and T2. Asterisk indicates a significant difference in the Bonferroni post hoc test.

	Total Reading Duration (s)		Durations of Fixations (ms)	
	T1	T2	T1	T2
G1	45.9 ± 5.15	39.9 ± 5.20	376 ± 23	330 ± 21
G2	51.9 ± 4.5	54.6 ± 5.1	296 ± 24	302 ± 25

ANOVA run on the number and amplitude of forward and backward saccades (see Table 3) reported a significant interaction T x G effect for the number of forward saccades only (F_(1,30)_ = 20.944, *p* < 0.001, η2 = 0.09), with a number of forward saccades significantly smaller for G1 at T2 (*p* = 0.05, Cohen’s d = 0.472).

## Data Availability

The datasets generated and/or analyzed during the current study are available from the author on reasonable request. The data is not publicly available due to ethical restrictions.
